# Serum, spleen metabolomics and gut microbiota reveals effect of catalpol on blood deficiency syndrome caused by cyclophosphamide and acetylphenylhydrazine

**DOI:** 10.3389/fimmu.2023.1280049

**Published:** 2023-11-03

**Authors:** Wensen Zhang, Na Cui, Fazhi Su, Yanping Sun, Biao Li, Yupeng Ren, Ping Wang, Haodong Bai, Wei Guan, Bingyou Yang, Qiuhong Wang, Haixue Kuang

**Affiliations:** ^1^ Key Laboratory of Basic and Application Research of Beiyao, Heilongjiang University of Chinese Medicine, Ministry of Education, Harbin, China; ^2^ Guangdong Engineering Technology Research Center for Standardized Processing of Chinese Materia Medica, Guangdong Pharmaceutical University, School of Chinese Materia Medica, Guangdong, China

**Keywords:** metabolomics, gut microbiota, catalpol, cyclophosphamide, rehmannia radix

## Abstract

Catalpol (CA), extracted from *Rehmannia Radix*, holds extensive promise as a natural medicinal compound. This study employed 16S rRNA gene sequencing and combined serum and spleen metabolomics to profoundly investigate the therapeutic effects of CA on blood deficiency syndrome (BDS) and the underlying mechanisms. Notably, CA exhibited effectiveness against BDS induced by cyclophosphamide (CP) and acetylphenylhydrazine (APH) in rats-CA substantially elevated levels of crucial indicators such as erythropoietin (EPO), granulocyte colony-stimulating factor (G-CSF), tumor necrosis factor-alpha (TNF-a), and interleukin-6 (IL-6). Additionally, CA could alleviate peripheral blood cytopenia. Furthermore, the analysis of 16S rRNA revealed that CA had the potential to reverse the Firmicutes/Bacteroidetes (F/B) ratio associated with BDS. Through comprehensive serum and spleen metabolomic profiling, we successfully identified 22 significant biomarkers in the serum and 23 in the spleen, respectively. Enrichment analysis underscored Glycerophospholipid metabolism and Sphingolipid metabolism as potential pathways through which CA exerts its therapeutic effects on BDS.

## 1 Introduction

Blood deficiency is a prevalent syndrome in clinical medicine, attributed to causes such as substantial blood loss, nutritional insufficiencies, inadequate hematogenesis, and iron depletion (1). In a modern medical context, it refers to the decrease in hemoglobin concentration and the blood pancytopenia. Traditional Chinese medicine (TCM) characterizes a pathological condition involving blood dysfunction and organ deterioration (2). Acetylphenylhydrazine (APH) is a potent oxidantthat gradually inflicts oxidative damage to red blood cells (RBC), leading to hemolytic anemia (3). In the field of cancer treatment, chemotherapy stands as the prevailing approach. Cyclophosphamide (CP), a broad-spectrum anticancer agent, exerts formidable cytotoxic effects on hematopoietic stem cells both in the bone marrow and circulating peripheral blood, culminating in anemia (due to hematopoietic inhibition) and compromised immune function (4). Commonly, CP, combined with antibiotic treatment, is used in cancer therapy due to the complications associated with bacterial translocation and infections caused by the mucosal barrier disruption (5). Chemotherapy alone alters the fecal microbiota in patients, including decrease species richness and absolute bacterial load (6). Consequently, an urgent need arises to identify a therapeutic agent capable of alleviating blood deficiency induced by chemotherapeutic interventions. A recent investigation established a hemolytic and aplastic anemia model using APH in combination with CP (7). This model closely mirrors the *in vivo* conditions characteristic of blood deficiency syndrome (BDS) in clinical scenarios.

*Rehmanniae Radix* (RR) is a TCM distributed in many provinces, such as Henan, Hebei, and Shanxi in China (8). Many clinical and experimental studies have shown that RR is an important TCM for treating bone loss by improving bone mineral density in patients with osteoporosis (9). RR’s steamed product, *Rehmanniae Radix* Praeparata (RRP), historically known for its “ nourish the bone marrow “ properties, has attracted contemporary attention for its bioactive ingredients, such as catalpol (CA; 10). Relevant pharmacokinetic studies have shown that CA can pass the blood-brain barrier, has the potential for oral administration, can be rapidly absorbed, and exhibits higher absolute bioavailability and a relatively long half-life (11). CA has many biological activities, such as antioxidant, anti-inflammatory, anticancer, antiapoptotic, and neuroprotective (12, 13).

In recent years, metabolomics has emerged as a powerful tool for understanding complex biological systems, offering valuable insights into metabolic pathways and their dynamic alterations. Concurrently, research into the gut microbiota has unveiled its pivotal role in human health and disease. An integrated analysis approach has emerged, fusing metabolomics and gut microbiota research to yield more profound and comprehensive insights. Metabolomics systematically studies small molecules, known as metabolites, within a biological system. These molecules are markers of biochemical processes and can provide a snapshot of an organism’s physiological state. Furthermore, integrated metabolomics and gut microbiota analysis offer promising avenues for personalized medicine. By elucidating individual-specific metabolic signatures linked to distinct microbiota compositions, clinicians can develop targeted interventions and therapies tailored to an individual’s unique physiological makeup.

## 2 Materials and methods

### 2.1 Chemicals and reagents

APH was provided by Aladdin; CP was provided by MACKLIN; Interleukin-6 (IL-6), Tumor Necrosis Factor-alpha (TNF-α), Erythropoietin (EPO), and Granulocyte Colony-Stimulating Factor (G-CSF) ELISA kit was provided by CUSABIO; CA was provided by Jingzhu bio-technology; Phospholipase D (PLD), Glyceraldehyde-3-Phosphate Dehydrogenase (GAPDH), and Sphingosine-1-Phosphate (S1P) as provided by Jiangsu Meimian Industrial Co., Ltd.

### 2.2 Animals and ethic statement

**Figure 1:**
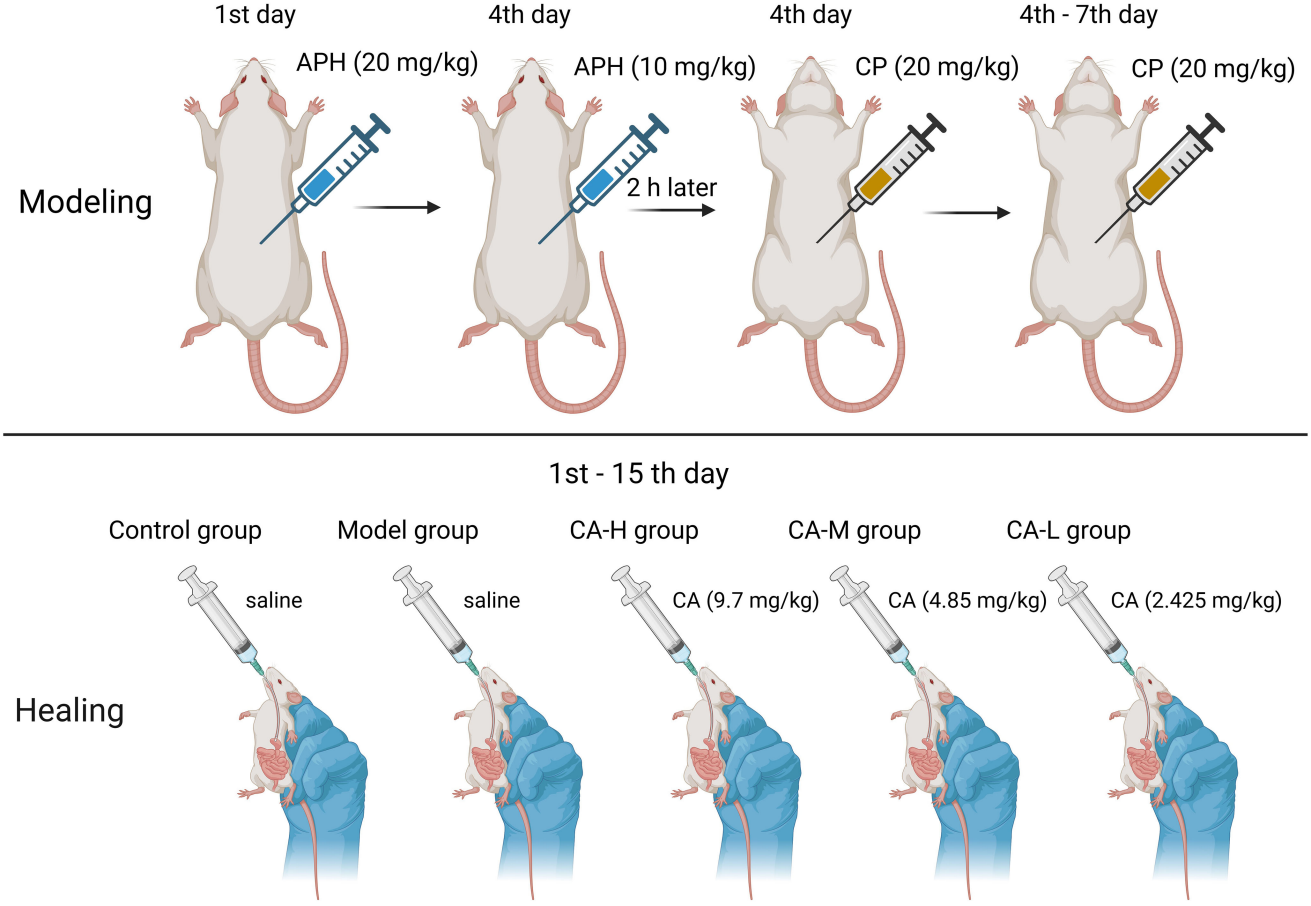
Modeling method in BDS rats.

Animal experiments were approved by the Ethics Committee of Heilongjiang University of Chinese Medicine (approval number: 2019121101) in this study. Forty male SD rats (180-220 g) were purchased from Guangdong Medical Experimental Animal Center (SCXK (Guangdong) 2017-0125). Rats breeding environment: temperature 24 ± 2°C, relative humidity 50 ± 2%, light and dark cycle 12-24 hours. After one week of adaptation, 40 rats were randomly divided into five groups: Control group; Model group; CA high dose (CA-H; 9.7 mg/kg) group; CA medium dose (CA-M; 4.85 mg/kg) group; CA low dose (CA-L; 2.425 mg/kg) group (14). The CA was reconfigured when it was used. Rats in the control and model groups were fed distilled water daily for 14 consecutive days. Except for the control group, rats in each group were subcutaneously injected with APH on day 1 (20 mg/kg) and day 4 (10 mg/kg). Two hours after the second APH injection, rats were intraperitoneally injected with CP (20 mg/kg) for four consecutive days. The control group received an equal volume of 0.9% saline following the same procedure (**Figure 1**).

### 2.3 Routine blood test

After being anesthetized with 20% uratan, blood was collected from the rats using vacuum blood collection tubes containing ethylenediaminetetraacetic acid (EDTA) to obtain plasma. We analyzed white blood cell (WBC), RBC, hematocrit (HCT), and hemoglobin (HGB) levels using a HEMAVET 950 automated hematology analyzer (Drew Scientific Group, Dallas, TX, USA).

### 2.4 ELISA kit detection

We collected rat blood using blood collection tubes without filler. The blood was allowed to stand at 4°C for 30 min and then centrifuged at 3500 rpm/min for 10 min. The serum was analyzed using an ELISA kit according to the manufacturer’s instructions, and the absorbance (OA) value was measured at 450 nm using a microplate reader. The levels of S1P, GAPDH, PLD, TNF-α, IL-6, EPO and G-CSF in serum were then calculated.

### 2.5 HE staining

The extent of spleen injury in rats was assessed using HE staining. Parts of the spleen tissue were fixed in 4% paraformaldehyde and embedded in paraffin. Paraffin blocks were cut into 10 μm thick sections with a microtome and stained with hematoxylin. The remaining unfixed spleen tissue was immediately transferred to liquid nitrogen and stored at -80°C for further analysis.

### 2.6 Metabolomics analysis

#### 2.6.1 Metabolomics sample preparation

Serum metabolomics: We mixed serum (100 µL) with acetonitrile (300 µL) and vortexed the mixture for 30 s. Then, the samples were placed in a high-speed centrifuge and centrifuged at 13500 rpm/min for 20 min at 4°C. Next, we collected 300 μL of the upper layer solution, which was evaporated and dried. Then, a mixture of 50% acetonitrile and water (150 µL) to redissolve the sample. Finally, we took 10 μL of each sample and pooled them together as a quality control sample for metabolomic analysis.

Spleen metabolomics: We weighed 100 mg of spleen tissue and added a mixed solution of 50% acetonitrile and water (300 μL). Placed into a tissue grinder and grind at 3500rpm/min for 3 min, repeat 3 times. Place the ground spleen tissue into a centrifuge and centrifuge at 13500 rpm/min for 15 min at 4°C. Then, the samples were placed in a high-speed centrifuge and centrifuged at 13500 rpm/min for 20 min at 4°C. Next, we collected 300 μL of the upper layer solution, which was evaporated and dried. Then, a mixture of 50% acetonitrile and water (150 µL) to redissolve the sample. Finally, we took 10 µL of each sample and pooled them together as a quality control sample for metabolomic analysis.

#### 2.6.2 Chromatography conditions

The treated samples were subjected to chromatography using a Waters ACQUITY UPLC HSS T3 column with gradient elution at a flow rate of 0.4 mL/min. The mobile phase A consisted of water containing 0.1% formic acid, while mobile phase B was acetonitrile containing 0.1% formic acid. The elution conditions for phase B were as follows: 0-2% (0-1 min), 2-35% (1-4 min), 35-100% (4-13 min), 100-100% (13-15.5 min), and 100-2% (15.5-19 min).

#### 2.6.3 MS conditions

We used an ultra-high performance liquid phase (Dionex Ultimate 3000, USA), samples were analyzed with a tandem electrostatic field orbitrap high-resolution mass spectrometer (Thermo orbitrap Fusion, USA). The spray voltage was set to +3.5 KV and −2.5 KV, respectively. The capillary voltage was set to 325°C, with an intrathecal gas volume flow rate of 50 arb and an auxiliary gas volume flow rate of 10 arb. The resolution was set to 120 000, and the acquisition range was m/z: 100-800. The HRMS2 intensity threshold was set to 2.0 × 10^4^. HRMS2 scanning high-energy collisional dissociation was set to 20%, 35%, 50% with a resolution of 30 000. Collision-induced dissociation was set to 15%, 30%, 45% with a resolution of 30 000.

#### 2.6.4 Biomarker identification and pathway analysis

Compound Discoverer 3.2 software was used for chromatographic peak identification, alignment, and normalization, and files containing m/z, retention time (Rt), and peak area were obtained. The original files were introduced into SIMCA 14.1 data processing software, and principal component analysis (PCA) and orthogonal partial least squares discriminant analysis (OPLS-DA) were used to analyze the rats in each group. Serum and spleen metabolites were analyzed. Based on the variable VIP > 1 and *p* < 0.05, the serum and spleen metabolites of rats in each group were compared to screen out potential biomarkers related to BDS. Based on the relative molecular mass and tandem mass spectrometry results, the mass spectrum information is matched with the mzCloud, Masslist Search, mzVault, and ChemSpider database to identify potential markers.

#### 2.6.5 DNA extraction and PCR amplification

Total microbial genomic DNA was extracted from colon contents samples using the PF Mag-Bind Stool DNA Kit (Omega Bio-tek, GA, USA) according to the manufacturer’s instructions. The quality and concentration of DNA were determined by 1.0% agarose gel electrophoresis and NanoDrop^®^ ND-2000 spectrophotometer (Thermo Scientific Inc., USA) and stored at −80°C before further use. The hypervariable region V3-V4 of the bacterial 16S rRNA gene was amplified by the ABI GeneAmp^®^ 9700 PCR thermal cycler using the primer pair 338F (5’-ACTCCTACGGGAGGCAGCAG-3’) and 806R (5’-GGACTACHVGGGTWTCTAAT-3’) (California, USA State ABI). The PCR reaction mixture included 4 μL 5 × Fast Pfu buffer, 2 μL 2.5 mM dNTPs, 0.8 μL forward and reverse primers (5 μM), 0.4 μL Fast Pfu polymerase, 10 ng template DNA, and ddH_2_O in a final volume of 20 µL. The PCR amplification cycle conditions were: initial denaturation at 95°C for 3 min, denaturation at 95°C for 30 s, annealing at 55°C for 30 s, extension at 72°C for 45 s, and 27 cycles of single extension. 72°C 10 min, end at 4°C. All samples were amplified in triplicate. PCR products were extracted and purified from 2% agarose gel. Quantification was then performed using a Quantus™ fluorometer (Promega, USA).

#### 2.6.6 Data processing

Raw FASTQ files were demultiplexed using an in-house perl script, then quality filtered via fastp version 0.19.6 and merged via FLASH version 1.2.11, meeting the following criteria: (i) Truncate 300 bp reads at any site with an average quality score < 20 on a 50 bp sliding window, and discard truncated reads shorter than 50 bp, and reads containing ambiguous characters are also discarded; (ii) Assemble based on overlapping sequences only Overlapping sequences longer than 10 bp. The maximum mismatch ratio in the overlapping area is 0.2. Reads that could not be assembled were discarded; (iii) Samples were distinguished based on barcodes and primers, and sequence directions were adjusted so that barcodes matched accurately and primers matched with 2 nucleotide mismatches. The optimized sequences were then clustered into operational taxonomic units (OTUs) using UPARSE 11 with a sequence similarity of 97%. The most abundant sequence for each OTU was selected as the representative sequence.

### 2.7 Statistical analysis

Based on OTU information, Mothur v1.30.2 was used to calculate rarefaction curves and alpha diversity indexes, including observed OTUs, Chao richness, Shannon index, and Simpson index. The similarity of microbial communities in different samples was determined by principal coordinate analysis (PCoA) based on Bray-curtis dissimilarity using the Vegan v2.4.3 package. The PERMANOVA test was used to assess the percentage of variation explained by treatment and its statistical significance using the Vegan v2.4.3 software package. Linear discriminant analysis (LDA) effect size (LEfSe) (http://huttenhower.sph.harvard.edu/LEfSe) was performed to identify taxa (phylum to genus) that were significantly enriched by bacteria in different groups (LDA score > 2, *p* < 0.05). Due to multicollinearity issues among clinical parameters, the variance inflation factor (VIF) of each variable was estimated using the vif function in the Vegan v2.4.3 package (https://cran.r-project.org/web/packages/car/car.pdf). Distance-based redundancy analysis (db-RDA) was performed using the Vegan v2.4.3 software package to study the impact of clinical parameters on intestinal bacterial community structure. Forward selection is based on a Monte Carlo permutation test (permutation = 9999). The values of the x- and y-axes and the length of the corresponding arrows represent the importance of each clinical parameter in explaining the distribution of taxa across communities. Linear regression analysis was applied to determine the association between the main clinical parameters determined by db-RDA analysis and the microbial alpha diversity index. The co-occurrence network is constructed to explore the internal community relationships between samples.

All data were expressed as mean ± standard deviation. A t-test was performed using GraphPad Prism 7 software (GraphPad Software, United States). A value of *p* < 0.05 was considered significant, while a value of *p* < 0.01 was considered highly significant. Histograms were generated using GraphPad Prism 7 software.

## 3 Results

### 3.1 Results of spleen coefficient

**Figure 2:**
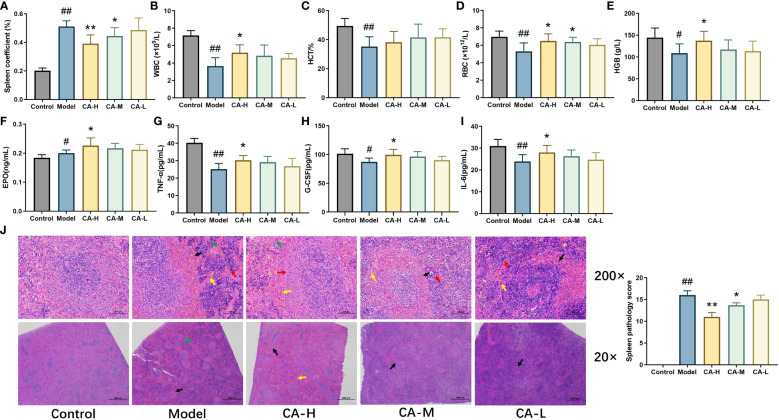
The effect of CA on physiological and biochemical indicators in BDS rats. **(A)** Rat spleen coefficient; **(B)** The level of WBC; **(C)** The level of HCT; **(D)** The level of RBC; **(E)** The level of HGB; **(F)** The level of EPO; **(G)** The level of TNF-α; **(H)** The level of G-CSF; **(I)** The level of IL-6; **(J)** HE staining (200 × and 20 ×), CA treatment of CP and APH induced spleen injury. Each value represents the mean ± SD (n = 3). ^##^ *p* < 0.01 or ^#^ *p* < 0.05, compared with the control group; *p < 0.05 or **p < 0.01, compared with the model group.

Compared with the control group, the model group exhibited a significant increase in spleen index (*p* < 0.01). Compared with the model group, the spleen index of the CA-H (*p* < 0.01) and CA-M (*p* < 0.05) groups significantly decreased. There was no significant difference in the spleen index of the CA-L group (**Figure 2A**). These findings suggest that the effect of CA on splenomegaly was dose-dependent.

### 3.2 Results of routine blood test

After 15 days of administration, we measured the rats’ WBC, RBC, HGB, and HCT levels (**Figures 2B–E**). Compared with the control group, the levels of RBC, WBC, HCT, and HGB were significantly decreased (*p* < 0.01) in the model group. Compared with the model group, the levels of WBC, RBC, and HCT in the CA-H group were significantly increased (*p* < 0.05), and the level of HGB was remarkably increased (*p* < 0.01).

### 3.3 Results of ELISA kit

We used ELISA kits to detect the levels of EPO, G-CSF, TNF-α, and IL-6 in serum. The data showed that compared with the control group, the levels of G-CSF, TNF-α, and IL-6 were significantly decreased (*p* < 0.05 or *p* < 0.01), and the level of EPO was increased (*p* < 0.05) in the model group. Compared with the model group, the levels of EPO, G-CSF, TNF-α, and IL-6 were significantly increased (*p* < 0.05 or *p* < 0.01) in the CA-H group, while in the CA-M group, the levels of TNF-α were significantly increased (*p* < 0.05) (**Figures 2F–I**).

### 3.4 Results of HE staining

Compared to the control group, the model group exhibited widespread white pulp atrophy with smaller cross-sections and loss of the peripheral marginal zone. At the same time, alongside peripheral capillary congestion, an increase in red blood cells (indicated by a black arrow), a moderate rise in extramedullary hematopoietic cells in the red pulp (red arrow), and more hemosiderin deposition (yellow arrow). Additionally, CP and APH expanded blood sinuses and increased red blood cells (green arrow) in the red pulp.

Compared with the model group, white pulp atrophy was mitigated in the CA-H group, and the cross-section was expanded (black arrow). The marginal zone around the white pulp was noticeable, broader, and relatively even in thickness (red arrow) compared to the model group. The red pulp demonstrated a reduction; in blood sinus congestion and lessened expansion, decreasing the number of red blood cells (yellow arrow). Moreover, there was a significant decrease in extramedullary hematopoietic cells, and less hemosiderin deposition was observable (green arrow; **Figure 2J**).

### 3.5 Results of metabolomics analysis

**Figure 3:**
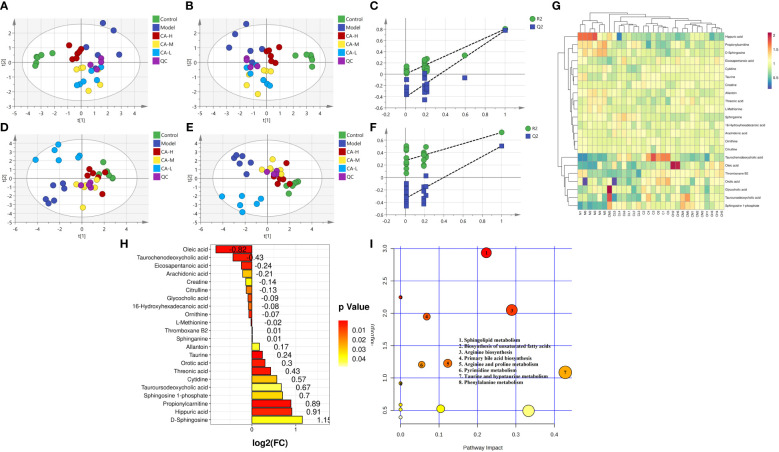
Serum metabolomics. **(A, D)** The PCA analysis represents the positive and negative ion modes of the ESI; **(B, E)** The PLS-DA analysis represents the positive and negative ion modes of the ESI (n = 6); **(C, F)** The permutation test analysis represents the positive and negative ion modes; **(G)** The heatmap and cluster analysis for the potential biomarker; **(H)** Folding change chart; **(I)** Bubble chart.

We employed an untargeted metabolomics approach to identify rat serum and spleen metabolites to investigate changes in host metabolism. Compounds detected in positive and negative modes used electrospray ionization (ESI+ and ESI−) in rat serum metabolism for multivariate statistical analysis. The QC samples are closely distributed and highly correlated, indicating that the entire detection process is stable. Principal component analysis (PCA) of ESI+ metabolites is shown in **Figure 3A**. The PCA plot shows that the serum samples of the control, model, CA-H, CA-M, and CA-L groups can be divided into clusters. Likewise, a clear separation was observed in the PLS-DA plot (**Figure 3B**). In order to verify the accuracy of PCA or PLS-DA, permutation test analysis (Q^2 = ^0.00461, R^2^ = -0.407, **Figure 3C**) was used, PCA and PLS-DA analysis of ESI− metabolites also showed significant differences among the control, model, CA-H, CA-M, and CA-L groups (**Figures 3D, E**). At VIP≥1.0, *p* < 0.05, 22 differential metabolites were obtained (**Table 1**; **Figure 3G**). The permutation test analysis (Q^2^ = 0.231, R^2^ = - 0.415, **Figure 3F**) was used. The fold change (FC) value is shown in **Figure 3H**. In addition, KEGG pathway enrichment analysis showed that in *p* < 0.05, differential metabolites were closely related to 4 metabolic pathways (**Figure 3I**), including Sphingolipid metabolism, Biosynthesis of unsaturated fatty acids, Arginine biosynthesis, and Primary bile acid biosynthesis.

**Table 1:** Detection of BDS related metabolites in serum metabolomics.

Metabolite	HMDB ID	Adducts	Formula	m/z	RT (min)	a	b	b	b
						Model	CA-H	CA-M	CA-L
Thromboxane B2	3252	M-H	C_20_H_34_O_6_	370.2364	7.15	↑	↑	↑	↑
Threonic acid	943	M-H	C_4_H_8_O_5_	136.0367	0.66	↑	↓	↓	↓
Tauroursodeoxycholic acid	874	M-H	C_26_H_45_NO_6_S	499.2977	7.23	↑	↓	↑	↑
Taurochenodeoxycholic acid	951	M-H	C_26_H_45_NO_6_S	499.2974	7.45	↓	↑	↑	↑
Taurine	251	M-H	C_2_H_7_NO_3_S	125.0142	0.62	↑	↓	↓	↓
Sphingosine 1-phosphate	277	M+H	C_18_H_38_NO_5_P	379.2511	8.95	↓	↑	↑	↑
Sphinganine	269	M+H	C_18_H_39_NO_2_	301.2995	8.74	↑	↓	↓	↓
Propionylcarnitine	824	M+H	C_10_H_19_NO_4_	217.1325	2.07	↑	↓	↓	↓
Orotic acid	226	M-H	C_5_H_4_N_2_O_4_	156.0166	0.79	↑	↑	↑	↑
Ornithine	214	M-H	C_5_H_12_N_2_O_2_	132.0894	0.63	↓	↑	↑	↑
Oleic acid	207	M-Na	C_18_H_34_O_2_	282.2564	13.88	↓	↑	↑	↑
L-Methionine	696	M+H	C_5_H_11_NO_2_S	149.0519	0.82	↓	↑	↓	↓
Hippuric acid	714	M-H	C_9_H_9_NO_3_	179.0578	4.44	↓	↓	↓	↓
Glycocholic acid	138	M-H	C_26_H_43_NO_6_	465.3099	7.08	↓	↓	↓	↓
Eicosapentanoic acid	1999	M-H	C_20_H_30_O_2_	302.2247	10.86	↓	↑	↑	↑
D-Sphingosine	252	M+H	C_18_H_37_NO_2_	299.2837	9.11	↑	↓	↓	↓
Cytidine	89	M+Na	C_9_H_13_N_3_O_5_	243.0869	0.79	↑	↓	↓	↓
Creatine	64	M-H	C_4_H_9_N_3_O_2_	131.0690	0.66	↓	↑	↑	↑
Citrulline	904	M-H	C_6_H_13_N_3_O_3_	175.0954	0.64	↓	↑	↑	↑
Arachidonic acid	1043	M-Na	C_20_H_32_O_2_	304.2409	12.98	↓	↓	↓	↓
Allantoin	462	M-H	C_4_H_6_N_4_O_3_	158.0435	0.66	↑	↓	↓	↓
16-Hydroxyhexadecanoic acid	6294	M-H	C_16_H_32_O_3_	272.2354	12.46	↓	↑	↑	↑

a, represents comparison with the control group; b, represents comparison with the model group. ↑, represents an increase in content; ↓, represents a decrease in content.

**Figure 4:**
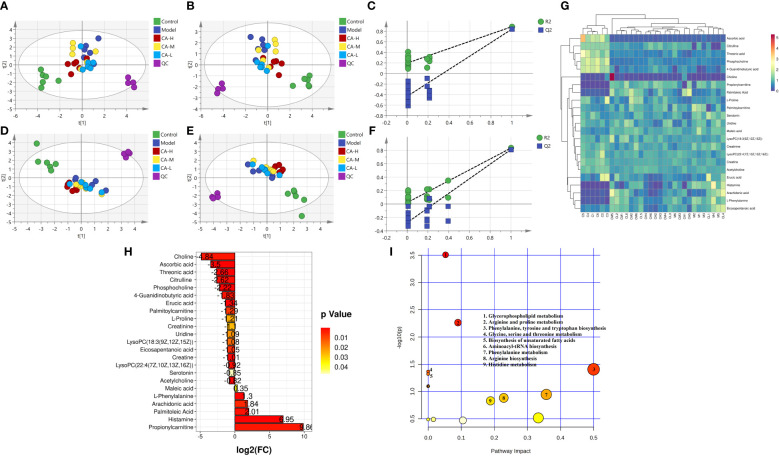
Spleen metabolomics. **(A, D)** The PCA analysis represents the positive and negative ion modes of the ESI; **(B, E)** The PLS-DA analysis represents the positive and negative ion modes of the ESI (n = 6); **(C, F)** The permutation test analysis represents the positive and negative ion modes; **(G)** The heatmap and cluster analysis for the potential biomarker; **(H)** Folding change chart; **(I)** Bubble chart.

Compounds detected in positive and negative modes used ESI+ and ESI− in rat spleen metabolism for multivariate statistical analysis. PCA of ESI+ metabolites was shown in **Figure 4A**. The PCA plot shows that the spleen samples of the control, model, CA-H, CA-M, and CA-L groups can be divided into clusters. Likewise, a clear separation was observed in the PLS-DA scoring scatter plot (**Figure 4B**). To verify the accuracy of PCA or PLS-DA, permutation test analysis (Q^2^ = 0.204, R^2^ = - 0.498, **Figure 4C**) was used. PCA and PLS-DA analysis of ESI− metabolites also showed significant differences among the control, model, CA-H, CA-M, and CA-L groups (**Figures 4D, E**). At VIP≥1.0, *p* < 0.05, 23 differential metabolites were obtained (**Table 2**; **Figure 4G**). The permutation test analysis (Q^2^ = 0.0206, R^2^ = - 0.277, **Figure 4F**) was used. The fold change (FC) value is shown in **Figure 4H**. In addition, KEGG pathway enrichment analysis showed that in *p* < 0.05, differential metabolites were closely related to 5 metabolic pathways (**Figure 4I**), including Glycerophospholipid metabolism, Arginine and proline metabolism, Phenylalanine, tyrosine and tryptophan biosynthesis, Glycine, serine and threonine metabolism, and Biosynthesis of unsaturated fatty acids.

**Table 2:** Detection of BDS related metabolites in spleen metabolomics.

Metabolite	HMDB ID	Adducts	Formula	m/z	RT (min)	a	b	b	b
						Model	CA-H	CA-M	CA-L
Serotonin	259	M+Na	C_10_H_12_N_2_O	159.0693	1.98	↓	↑	↑	↑
Propionylcarnitine	824	M+H	C_10_H_19_NO_4_	217.1332	1.85	↑	↓	↓	↓
Phosphocholine	1565	M+Na	C_5_H_14_NO_4_P	183.0668	0.61	↓	↓	↓	↓
Palmitoylcarnitine	222	M+H	C_23_H_45_NO_4_	399.3368	10.29	↓	↑	↑	↑
Palmitoleic Acid	3229	M+H	C_16_H_30_O_2_	276.2103	10.71	↑	↓	↓	↓
LysoPC(22:4(7Z,10Z,13Z,16Z))	10401	M+H	C_30_H_54_NO_7_P	571.3669	10.58	↓	↑	↑	↑
LysoPC(18:3(9Z,12Z,15Z))	10388	M+H	C_26_H_48_NO_7_P	517.3185	9.02	↓	↑	↑	↑
L-Proline	162	M+H	C_5_H_9_NO_2_	115.0636	5.18	↓	↑	↓	↓
Histamine	870	M+H	C_5_H_9_N_3_	111.0807	0.51	↑	↑	↑	↑
Creatinine	562	M+H	C_4_H_7_N_3_O	113.0592	0.77	↓	↓	↓	↓
Choline	97	M+H	C_5_H_13_NO	103.0998	6.34	↓	↑	↓	↓
Acetylcholine	895	M+H	C_7_H_15_NO_2_	145.1108	0.66	↓	↓	↓	↓
4-Guanidinobutyric acid	3464	M+H	C_5_H_11_N_3_O_2_	145.0854	0.81	↓	↓	↓	↓
Citrulline	904	M+H	C_6_H_13_N_3_O_3_	175.0962	0.62	↓	↓	↓	↓
Uridine	296	M-H	C_9_H_12_N_2_O_6_	244.0700	1.44	↓	↑	↑	↑
Threonic acid	943	M-H	C_4_H_8_O_5_	136.0376	0.69	↓	↑	↑	↑
Maleic acid	176	M-H	C_4_H_4_O_4_	116.0105	1.29	↓	↑	↑	↑
L-Phenylalanine	159	M-H	C_9_H_11_NO_2_	165.0785	3.10	↑	↑	↑	↑
Erucic acid	2068	M-H	C_22_H_42_O_2_	338.3191	15.88	↓	↑	↑	↑
Eicosapentanoic acid	1999	M-H	C_20_H_30_O_2_	302.2249	12.34	↓	↑	↑	↑
Creatine	64	M-H	C_4_H_9_N_3_O_2_	131.0691	0.70	↓	↓	↓	↓
Ascorbic acid	44	M-Na	C_6_H_8_O_6_	116.0107	0.73	↓	↓	↓	↓
Arachidonic acid	1043	M-Na	C_20_H_32_O_2_	304.2410	12.98	↑	↑	↑	↑

a, represents comparison with the Control group; b, represents comparison with the Model group. ↑, represents an increase in content; ↓, represents a decrease in content.

### 3.6 Results of gut microbiota analysis

#### 3.6.1 The effects of CA on the structure of the gut microbiota

**Figure 5:**
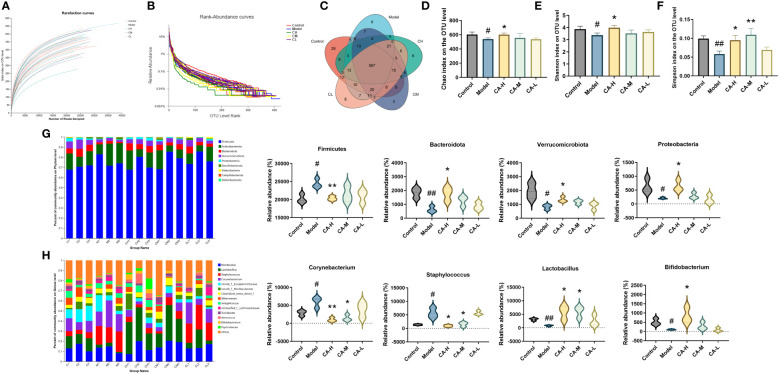
Effect of CA on the richness and diversity of colonic contents microbiota in rats. **(A)** Rarefaction curves. **(B)** Rank abundance curves. **(C)** OTU Venn diagram. **(D)** Chao index. **(E)** Simpson index. **(F)** Shannon index. **(G, H)** The colonic contents bacterial community at the phylum, and genus levels. Less than 0.5% abundance of the genus was merged into others. Each value represents the mean ± SD (n = 3). ^#^ *p* < 0.05 or ^##^ *p* < 0.01, compared with the control group; *p < 0.05 or **p < 0.01, compared with the model group.

To investigate the impact of CA on the gut microbiota of BDS rats, we performed 16s RNA sequencing on the colonic contents of control, model, CA-H, CA-M, and CA-L groups rats. The sequencing reads were sufficient for subsequent analysis, as shown in **Figure 5** (n = 3), with 687403 sequencing reads obtained from 15 samples. After removing ineligible sequences, Venn diagrams were used to represent the characteristics of different treatment groups and common taxonomic groups. Based on > 97% similarity between sequences, 567 OTUs in the gut microbiota of all groups (**Figure 5C**). The species diversity of each sample was assessed using the alpha diversity statistical analysis index. The rarefaction curves (Sobs index at the OTU level) tended to be stable (**Figure 5A**), and the rank-abundance curves tended to be smooth (**Figure 5B**). The Chao, Simpson, and Shannon indexes were negatively correlated with diversity. The model group’s Shannon, Shannon, and Chao indexes were significantly lower than the control group. However, the Shannon, Simpson, and Chao indexes were significantly higher in the CA-H group compared with the model group (**Figures 5D–F**).

The heatmap plots of the dominant gut microbiota composition at the phylum level (**Figure S1A**) and the genus level (**Figure S1B**) reveal variations in the abundance of prominent species through an analysis of the gut microbiota. Compared with the model group, the relative abundance of *Proteobacteria*, *Bacteroidota*, and *Verrucomicrobiota* is significantly increased in the CA-H group, while the relative abundance of *Firmicutes* at the phylum level is notably decreased. When compared to the model group, the CA-H group shows a substantial increase in the relative abundance of *Lactobacillus* and *Bifidobacterium*, accompanied by a significant decrease in the relative abundance of *Corynebacterium* and *Staphylococcus* at the genus level (**Figures 5G, H**).

**Figure 6:**
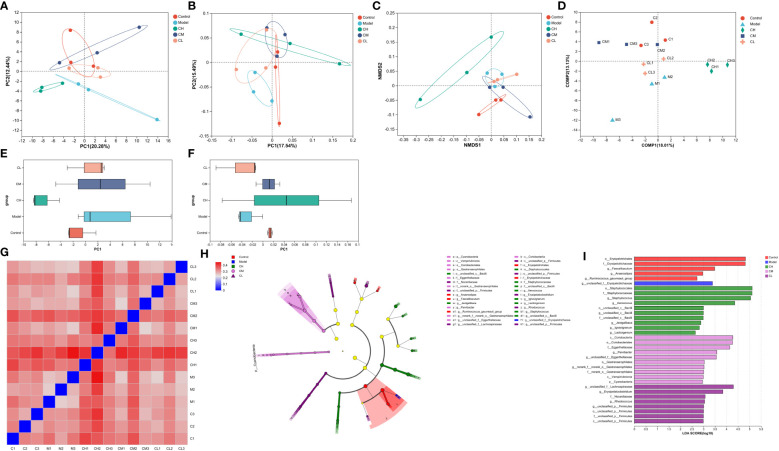
β-diversity analysis of gut microbiota. **(A)** PCA analysis; **(B)** PCoA analysis; **(C)** NMDS analysis; **(D)** PLS-DA analysis; **(E)** PCA box diagram; **(F)** PCoA box diagram; **(G)** β-diversity heatmap; **(H)** The indicator bacteria with an LDA score of 2 or higher in the bacterial community were associated with the five groups of rats; **(I)** The areas with different colors represent different components (red, control group; blue, model group; green, CA-H group; pink, CA-M group; purple, CA-L group). The circle indicates the level of phylogeny from phylum to genus.

Based on unweighted uniFrac distances, we utilized PCoA analysis to assess gut microbiota diversity in BDS rats. The outcomes revealed significant distinctions across the five groups (**Figures 6B, F**), indicative of gut microbial dysbiosis in BDS rats. Corresponding observations were evident in the NMDS analysis (**Figure 6C**). The PCA plot featured MetaStats analysis of the sequencing data (**Figures 6A, E**), aligning with the findings from PCoA and NMDS analyses. PLS-DA analysis (**Figure 6D**) enhanced these results and illustrated improved group dispersion. We generated a heatmap (**Figure 6G**) rooted in the unweighted uniFrac distances between samples to provide additional validation. This heatmap highlighted the substantial treatment effect of BDS within the CA-H group, surpassing that of the CA-M group, while the influence in the CA-M group exceeded that in the CA-L group.

We employed the linear discriminant analysis effect size (LEfSe) technique to discern specific bacterial taxa within distinct populations. This method can analyze data from various branches of a microbial community. Due to the extensive dataset, comprehensive statistical analyses were undertaken from the phylum to the genus level, given the abundance of operational taxonomic units (OTUs) detected in this study. The arrangement presented in the cladograms, with an LDA score of 2 or higher, was confirmed to indicate significant differences (**Figure 6H**). The outcomes showcased in **Figure 6I** spotlight the bacteria displaying the most pronounced distinctions among the groups: *o_Erysipelotrichales* and *g_unclassified_f:Erysipelotrichaceae* in the control group, *o_Staphylococcales* in the model group, *c_Coriobacteriia* in the CA-H group, and *g_unclassified_f:Lachnospiraceae* in the CA-L group.

#### 3.6.2 Annotation of the gut microbiota function

**Figure 7:**
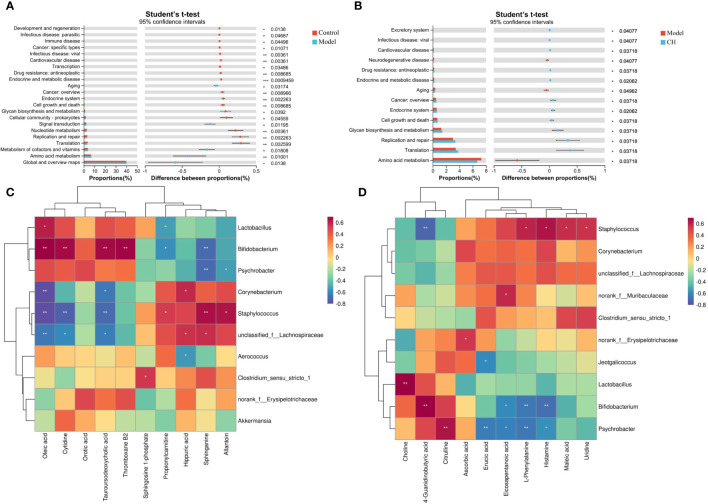
Functional prediction of altered gut microbiota by PICRUSt2 analysis based on the rat level II KEGG pathway. **(A)** Control group vs model group; **(B)** Model group vs CA-H group. **(C)** Spearman correlation heatmap between the top 10 important serum metabolite concentrations and the relative abundance of gut microbiota genus levels in the CA-H group; **(D)** The top 10 important spleen essential metabolites and gut microbiota in the CA-H group relative abundance at the genus level. R values are represented by gradient colors, where red and blue cells indicate positive and negative correlations. *p < 0.05 or **p < 0.01.

To elucidate the functional roles of the gut microbiota, we employed PICRUSt2 functional predictions, which involve species-level predictions and their functional relationships based on the KEGG database. We presented the results from a t-test analysis, comparing the predicted functional changes in the gut microbiota across different groups: control vs. model groups and model vs. CA-H groups. There were notable increases in several functional categories compared to the model group. Specifically, functions related to Translation, Metabolism of cofactors and vitamins, Amino acid metabolism, Global and overview maps, and others exhibited significantly higher levels in the control group (**Figure 7A**). These findings suggest that the control group’s gut microbiota may be associated with a more active and diverse functional process in these areas. Turning to the comparison between the model and CA-H groups, the CA-H group displayed considerably elevated levels in various functional categories when contrasted with the model group. Significant alterations were observed in Glycan biosynthesis and metabolism, Replication and repair, Translation, Amino acid metabolism, and other areas (**Figure 7B**). These results suggest that the CA-H group’s gut microbiota may contribute to enhanced functional activities in these pathways compared to the model group.

### 3.7 Correlation among gut microbiota and metabolic parameters in rat

The research team conducted a Spearman correlation analysis on 22 common serum metabolites and gut microbiota at the genus level, which can further analyze the interaction between gut microbiota and serum metabolites. *Staphylococcus* was positively correlated with Sphinganine, Propionylcarnitine, and Allantoin; Tauroursodeoxycholic acid, Oleic acid, and Cytidine were negatively correlated. *Corynebacterium* was positively correlated with Hippuric acid; Tauroursodeoxycholic acid, Oleic acid, and Eicosapentanoic acid were negatively correlated. Hippuric acid, and Allantoin were negatively correlated. *Bifidobacterium* was positively correlated with Thromboxane B2, Tauroursodeoxycholic acid, Oleic acid, and Cytidine; Sphinganine and propionylcarnitine were negatively correlated (**Figure 7C**). We performed a Spearman correlation analysis of 23 shared spleen metabolites with genus-level gut microbiota. *Psychrobacter* was positively correlated with Citrulline; *Psychrobacter* was negatively correlated with L-Phenylalanine, Histamine, Erucic acid, Eicosapentanoic acid, and Arachidonic acid. *Staphylococcus* was positively correlated with Urine, Maleic acid, L-Phenylalanine, and Histamine; 4-Guanidinobutyric acid was negatively correlated. *Bifidobacterium* was positively correlated with 4-Guanidinobutyric acid; L-Phenylalanine, Histamine, and Eicosapentanoic acid were negatively correlated (**Figure 7D**).

### 3.8 Results of expression of key targets

**Figure 8:**
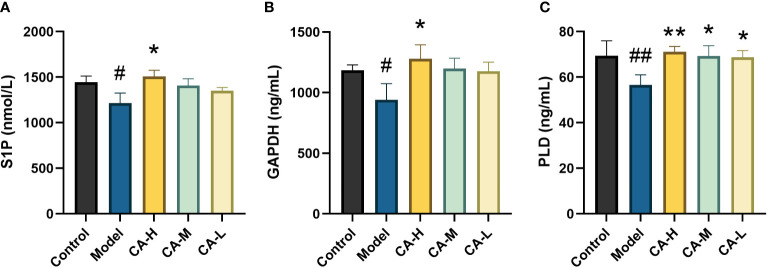
Effect of each group on the key target expression. **(A)** The level of S1P; **(B)** The level of GAPDH; **(C)** The level of PLD. ^##^ *p* < 0.01 or^#^ *p* < 0.05, compared with the control group; *p < 0.05 and **p < 0.01, compared with the model group.

Compared with the control group, the expression of S1P, GAPDH, and PLD in the spleen of the model group was decreased (*p* < 0.05 or *p* < 0.01). Compared with the model group, the CA-H group had increased expression of S1P, GAPDH, and PLD (*p* < 0.05 or *p* < 0.01), and the CA-M and CA-L groups had significantly increased expression of PLD (*p* < 0.01) (**Figure 8**).

## 4 Discussion

Chemotherapy, a cornerstone of cancer treatment, employs potent agents such as CP to combat cancer cells (15). While offering promising therapeutic outcomes, this treatment strategy can also introduce a range of potential challenges that demand careful consideration. A significant concern associated with chemotherapy is BDS, which hinders the bone marrow’s capacity to produce essential blood cells (16). This suppression often results in anemia, reduced RBC and WBC, leading to fatigue and weakness (17). Chemotherapy can trigger hair loss (18). Additionally, chemotherapy can compromise the immune system, making individuals more susceptible to infections and illnesses. While chemotherapy holds the potential to save lives by targeting and eradicating cancer cells, its adverse effects are an inherent part of the treatment process. Therefore, solving the side effects caused by CP and APH is imminent. CA is primarily utilized for specific immune therapy (19). As a result, compared with the model group, upon administering CA to rats, the spleen index of the CA-H group exhibited a notable decrease. The blood routine levels were also observed, revealing that HCT, RBC, WBC, and HGB levels in the CA-H group showed significant improvements. Histopathological analysis indicated that the rats in the CA-H group experienced significant enhancement in spleen morphology. However, no significant changes were observed in the CA-L group compared to the model group.

EPO and G-CSF are two hematopoietic growth factors that play essential roles in regulating blood cell production in the body (20). EPO’s prominent role is to stimulate the production of RBC in the kidney. EPO helps maintain the appropriate level of hemoglobin and RBC, ensuring effective oxygen delivery to tissues and organs (21). G-CSF is another growth factor that stimulates the production of specific WBCs called granulocytes, particularly neutrophils (22). Neutrophils are crucial for the immune response against bacterial infections (23). While G-CSF is not directly related to RBC production, its role in maintaining the immune system is essential. After treatment, the CA-H group showed significant differences from the model group (*p* < 0.05). CA promoted the restoration of RBC production by promoting the synthesis and secretion of EPO in rat bone marrow. The results in **Figure 2H** showed that APH and CP significantly inhibited G-CSF expression, which was sharply increased after CA administration. It suggested that CA had a specific inhibitory effect on the cytotoxicity of CP and APH. Increase the level of G-CSF and promote the recovery of granulocyte hematopoietic function. Immunocytokines such as TNF-α and IL-6 play an essential role in the coordination of the immune system (24). They enable the communication of lymphatic, inflammatory, and hematopoietic cells. In this study, serum TNF-α and IL-6 (**Figures 2G, I**) levels were significantly lower in the BDS group compared with the control group. Interestingly, CA administration reversed this inhibitory effect, suggesting its potential to enhance immune function and alleviate BDS.

In recent years, gut microbiota has received extensive attention as an important target for immune system diseases. Under normal conditions, the gut microbiota, consisting of billions of bacteria, maintains a symbiotic relationship with its host and helps regulate the body’s metabolism and energy. Many studies have shown that the changes in the gut microbiota of immune system diseases mainly increase the number of *Firmicutes* and reduce the proportion of *Bacteroidetes*. *Firmicutes* to *Bacteroidetes* (F/B) ratio has become one of the most important reference indicators for studying gut microbiota disorders (25). According to 16S rRNA sequencing, CA enhanced gut microbiota composition in BDS model rats, decreased the abundance of *Firmicutes*, and increased the abundance of *Bacteroidetes*. At the genus level, taking CA tended to stimulate the emergence of some potential probiotics, such as *Bifidobacterium* and *Lactobacillus*. Reduce the abundance of potentially harmful bacteria such as *Corynebacterium* and *Staphylococcus*. CA treatment reversed most of the CP and APH induced changes in the gut microbiota, suggesting that CA helps restore CP and APH induced changes in the interplay between the gut microbiota and immunity.

Our observations also demonstrated that the dysregulation of hematopoiesis is associated with Glycerophospholipid metabolism and Sphingolipid metabolism. This includes LysoPC(22:4(7Z,10Z,13Z,16Z)), LysoPC(18:3(9Z,12Z,15Z)), Choline, Acetylcholine, Sphingosine, Sphinganine, and Sphingosine-1-phosphate (S1P). Orsini’s study provides evidence of sphingolipids participating in TNF-α-mediated modulation of the TF/miR network and inhibition of autophagy in HSPCs, affecting erythrocyte formation (26). Sphinganine is a derivative of Sphingosine. The accumulation of these molecules has been confirmed to suppress Bcl-xL expression and downregulate Bcl-2, thereby enhancing apoptosis, accompanied by robust inhibition of the MAPK cascade (27–30). Additionally, Vu’s study indicated that a significant decrease in plasma S1P levels in Major facilitator superfamily transporter 2b (Mfsd2b) knockout mice results in damaged erythrocyte membranes and disordered peripheral blood (31). They also discovered that Sphingosine accumulates significantly when the secretion of S1P is blocked due to Mfsd2b knockout in RBC. In contrast, S1P, synthesized through the phosphorylation of Sphingosine in the presence of sphingosine kinase 1 (SphK1), has been shown to possess antiapoptotic properties (30). Our research, along with the work of Wątek, demonstrates a significant up-regulation in the levels of Sphingosine and Sphinganine in the model with immune damage mediated by chemotherapeutic drugs (32). Simultaneously, the concentration of S1P is notably lower (*p* < 0.05). Following CA treatment, there is a substantial decrease in the attention of Sphingosine and Sphinganine compared to the model (*p* < 0.05). Moreover, S1P levels increase significantly after CA treatment (*p* < 0.05). Phospholipase D (PLD) is an enzyme essential for immune receptor signaling and immune cell function. It catalyzes the hydrolysis of phosphatidylcholine, the major phospholipid in the plasma membrane, producing the critical signaling lipid phosphatidic acid (33). In addition to its role in immune cell function, PLD has been shown to play a role in Fcγ-mediated phagocytosis of macrophages and in activating NADPH oxidase in neutrophils (34). Coffman’s research reported that tumor growth factor-β (TGF-β) enhances the production of immunoglobulin (Ig) A by lipopolysaccharide-stimulated murine B lymphocytes. In this study, glyceraldehyde-3-phosphate dehydrogenase (GAPDH) significantly increased total IgA levels in human peripheral blood mononuclear cells (PBMC), mouse serum, and spleen lymphocytes (35). Additionally, Kinoshita’s research revealed that cell surface GAPDH plays a role in the adhesion of lactic acid bacteria to human mucin in the intestine. These findings suggest that GAPDH or its peptides may have immunomodulatory effects on the blood and intestinal immune systems (36). Furthermore, sphingolipids are vital signaling molecules that regulate numerous cellular processes crucial for immunity, inflammation, infection, and cancer. They serve as the fundamental building blocks of eukaryotic cell membranes. These sphingolipid metabolites are ceramide, ceramide-1-phosphate, sphingosine, and sphingosine-1-phosphate (S1P; 37, 38).

In this study, significant decreases (*p* < 0.05) were observed in the spleen levels of LysoPC(22:4(7Z,10Z,13Z,16Z)) and LysoPC(18:3(9Z,12Z,15Z)) 37. However, after CA treatment, these indexes were notably restored (*p* < 0.05). Abundant lysophosphatidylcholine (LPC) in plasma has been confirmed to contribute to the activation of inflammatory responses (39). We speculate that CA might exert its effects by suppressing inflammation and oxidative damage by restoring LPC levels. Additionally, CA could promote Glycerophospholipid metabolism, thus creating a more favorable hematopoietic microenvironment for enhancing hematopoiesis.

## 5 Conclusion

In summary, our study revealed multiple ameliorative effects of CA on BDS, including improving spleen function, enhancing immune levels, and pathological changes. The mechanism of CA treating BDS may be related to improving intestinal flora imbalance and regulating sphingolipid metabolism and glycerophospholipid metabolism.

## Data availability statement

The datasets presented in this study can be found in online repositories. The names of the repository/repositories and accession number(s) can be found here: NCBI, PRJNA1010515.

## Ethics statement

The animal study was approved by Ethics Committee of Heilongjiang University of Chinese Medicine (approval number: 2019121101). The study was conducted in accordance with the local legislation and institutional requirements.

## Author contributions

WZ: Writing – original draft. NC: Writing – review & editing. FS: Validation. YS: Formal analysis. BL: Formal analysis. YR: Validation. PW: Validation. HB: Validation. WG: Validation. BY: Supervision, Project administration. QW: Supervision, Project administration. HK: Supervision, Project administration.
