# Supplementary material for: Serum, spleen metabolomics and gut microbiota reveals effect of catalpol on blood deficiency syndrome caused by cyclophosphamide and acetylphenylhydrazine
Source: Front Immunol. 2023 Nov 3;14:1280049. doi: 10.3389/fimmu.2023.1280049 (PMC10655121; doi:10.3389/fimmu.2023.1280049)
Supplement: Supplementary file 1 [file DataSheet_1.zip › Supplementary_Material.docx]

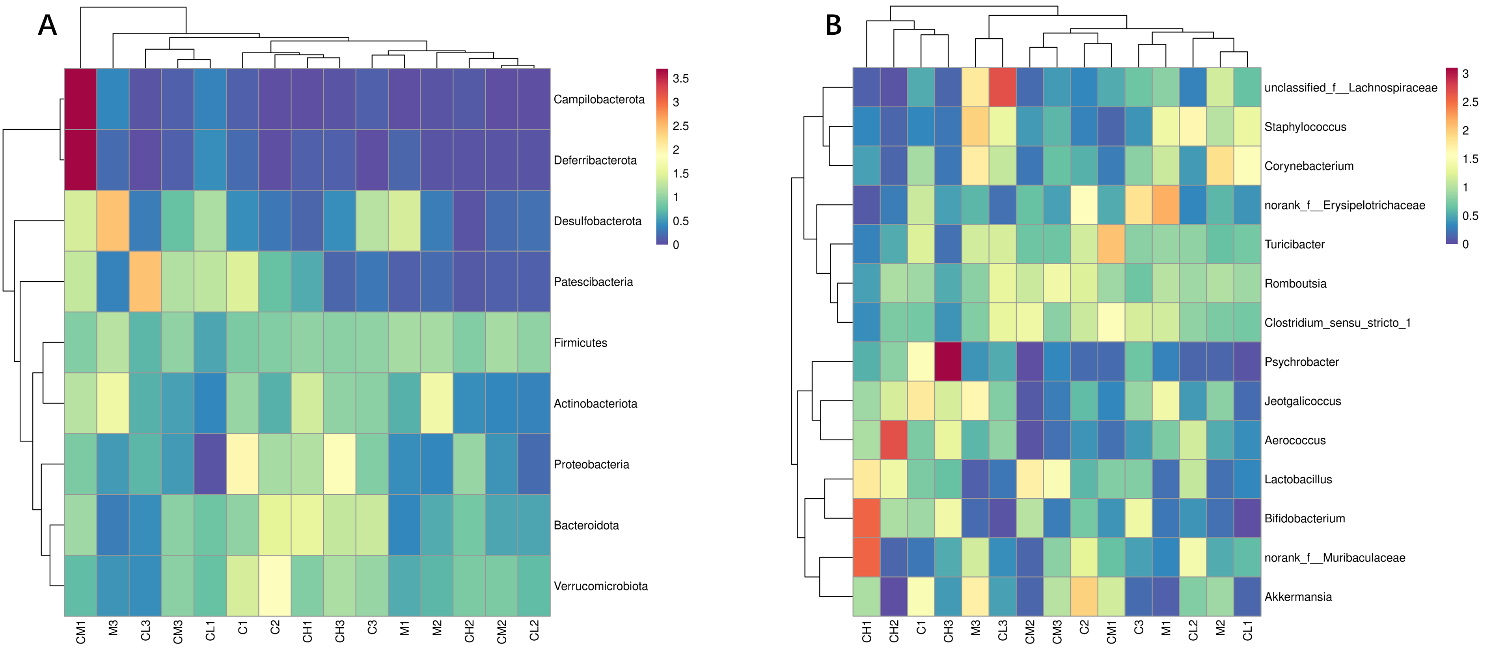


**Supplementary Figure 1.** The heatmap plots of the dominant microbial flora composition at the phylum level (A) and the genus level (B).
