# Supplementary figures and images for: Serum, spleen metabolomics and gut microbiota reveals effect of catalpol on blood deficiency syndrome caused by cyclophosphamide and acetylphenylhydrazine
Source: Front Immunol. 2023 Nov 3;14:1280049. doi: 10.3389/fimmu.2023.1280049 (PMC10655121; doi:10.3389/fimmu.2023.1280049)

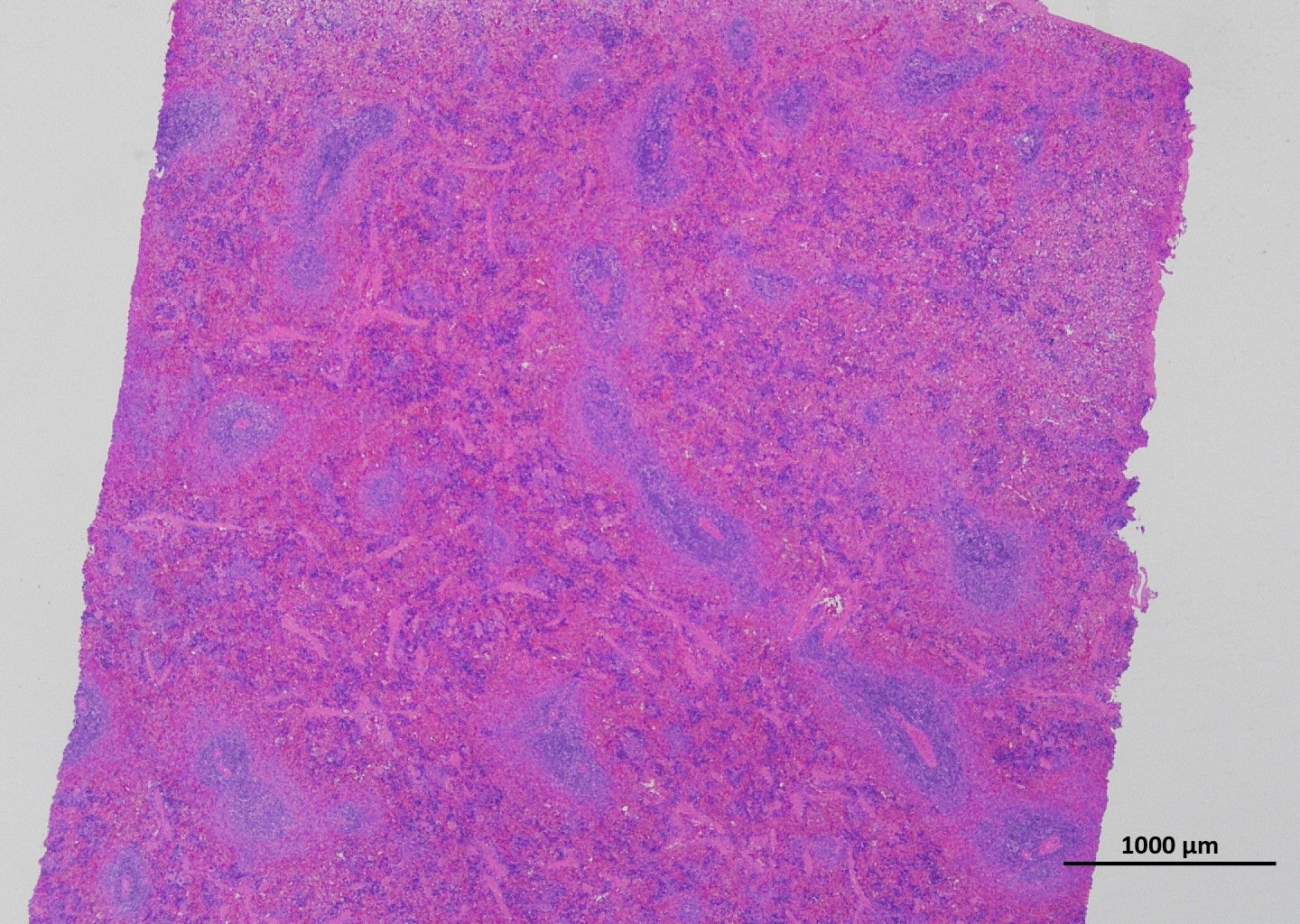

Supplement: Supplementary file 1 [file DataSheet_1.zip › HE/CA-H-20.jpg]

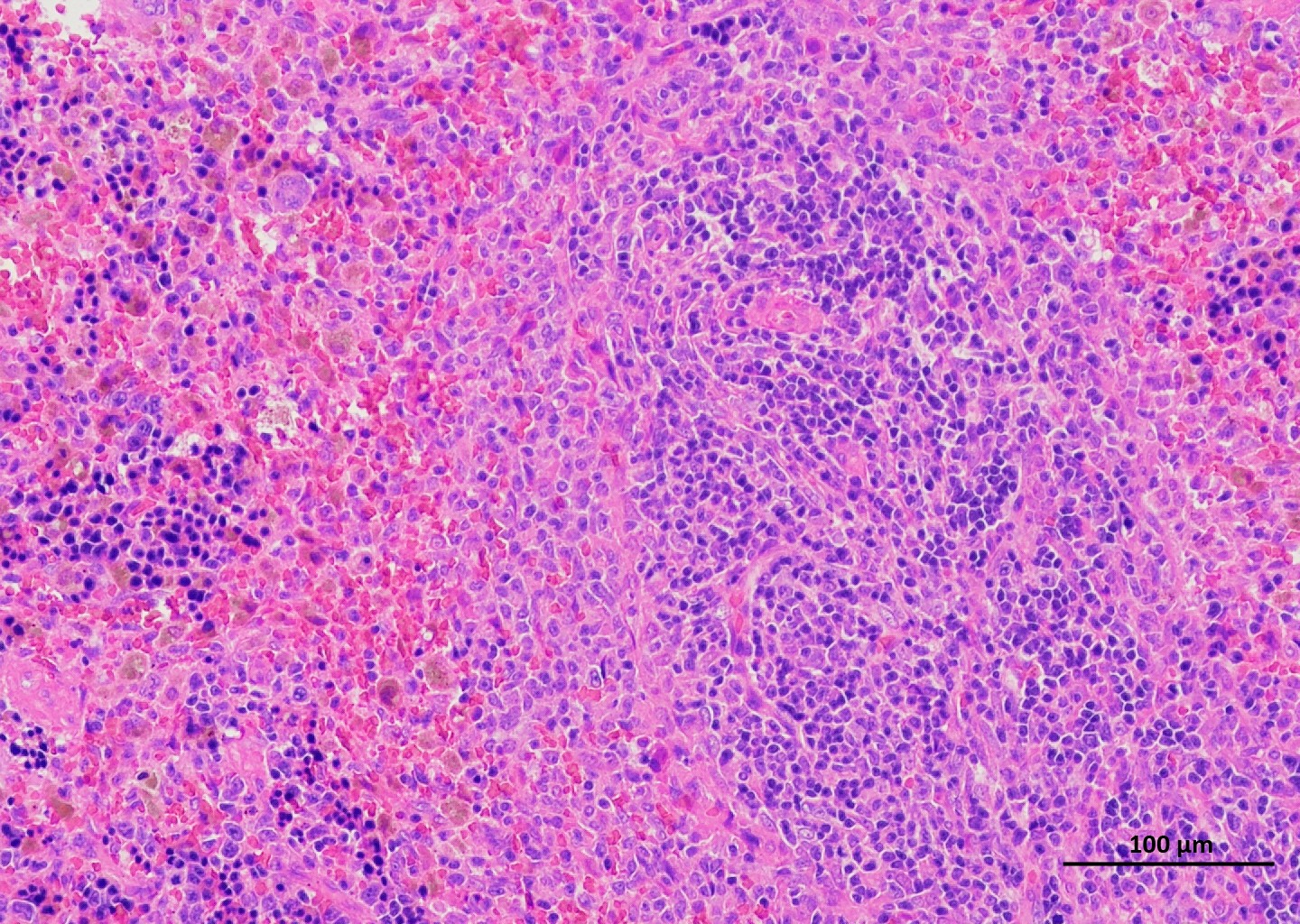

Supplement: Supplementary file 1 [file DataSheet_1.zip › HE/CA-H-200.jpg]

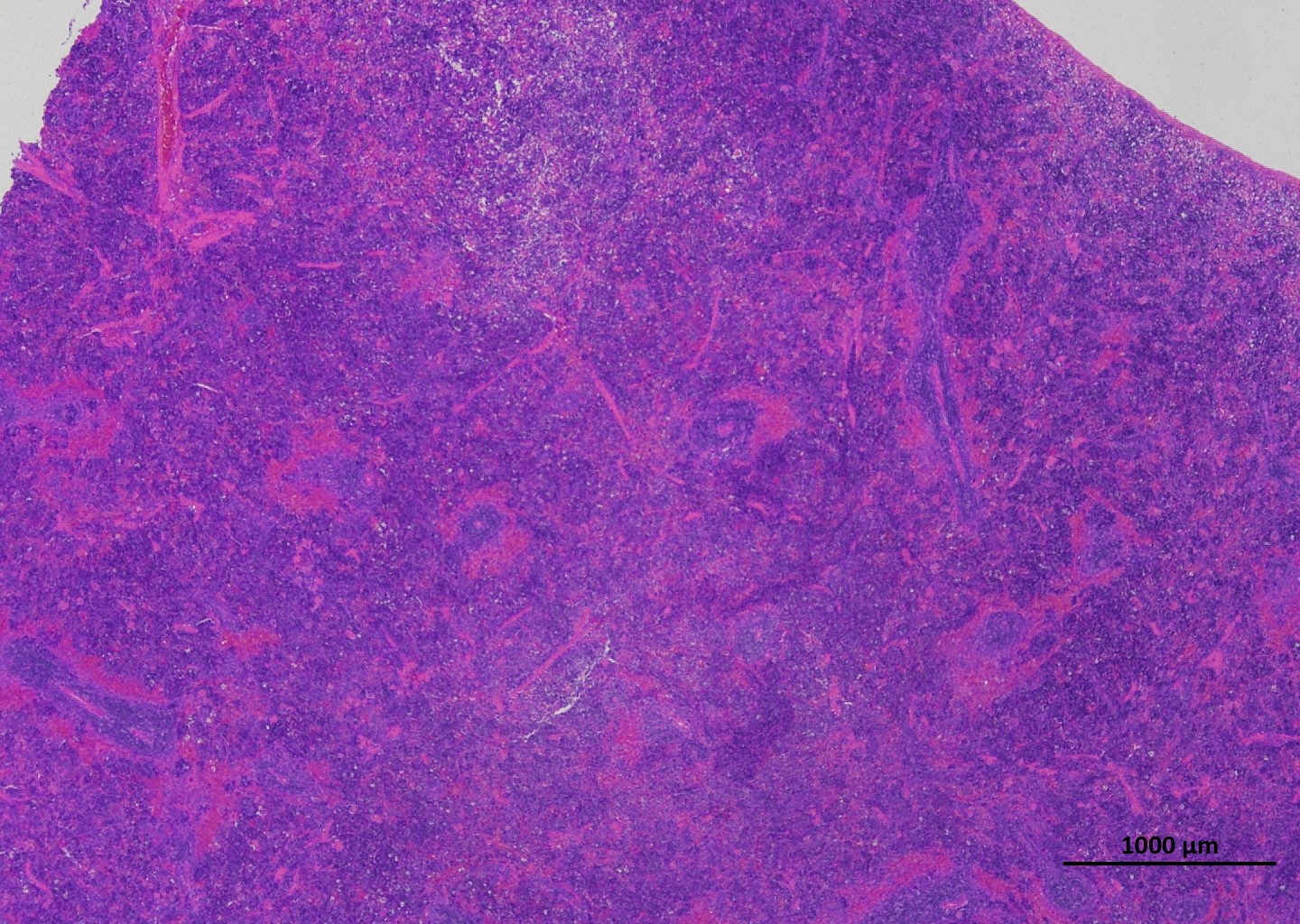

Supplement: Supplementary file 1 [file DataSheet_1.zip › HE/CA-L-20.jpg]

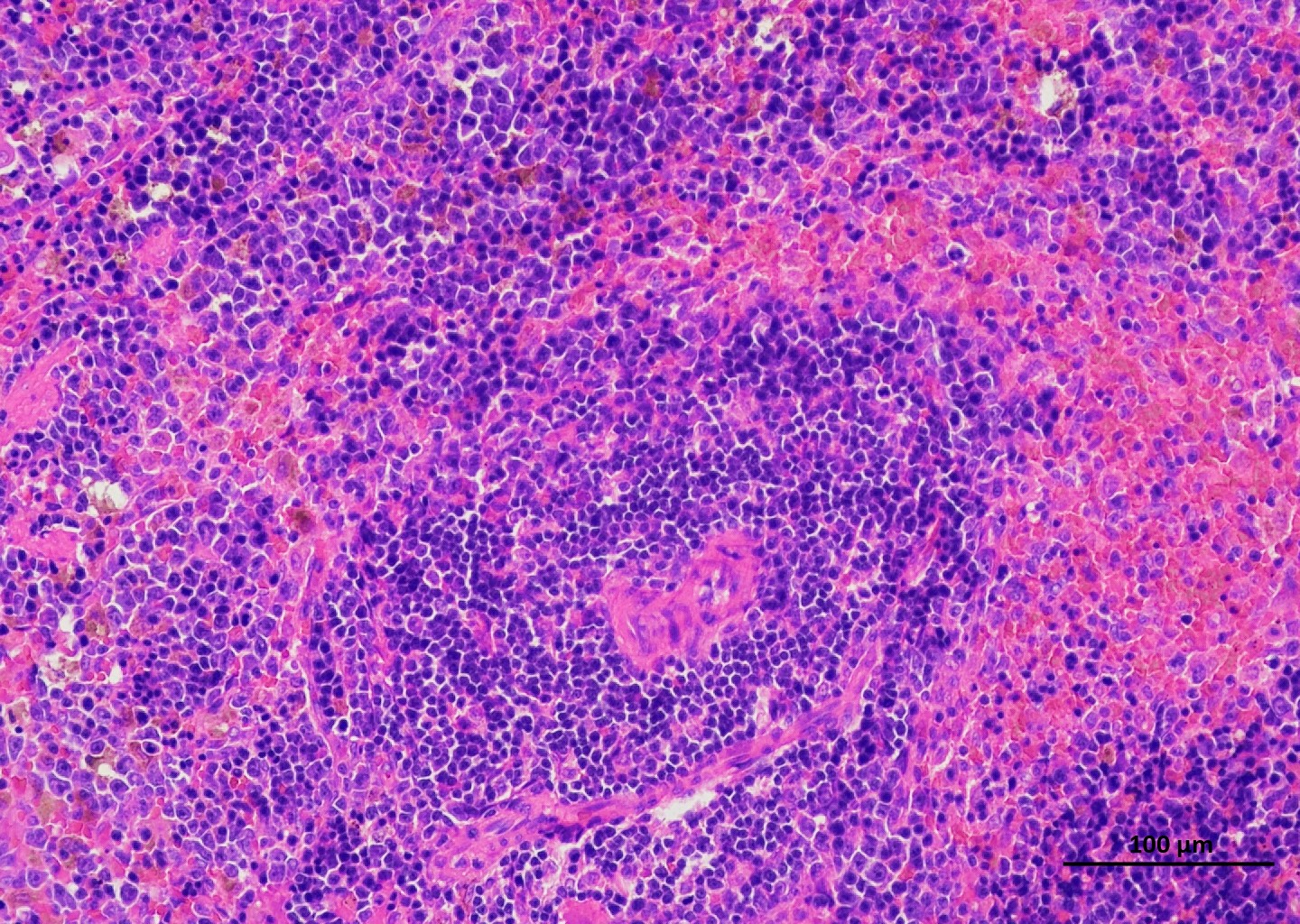

Supplement: Supplementary file 1 [file DataSheet_1.zip › HE/CA-L-200.jpg]

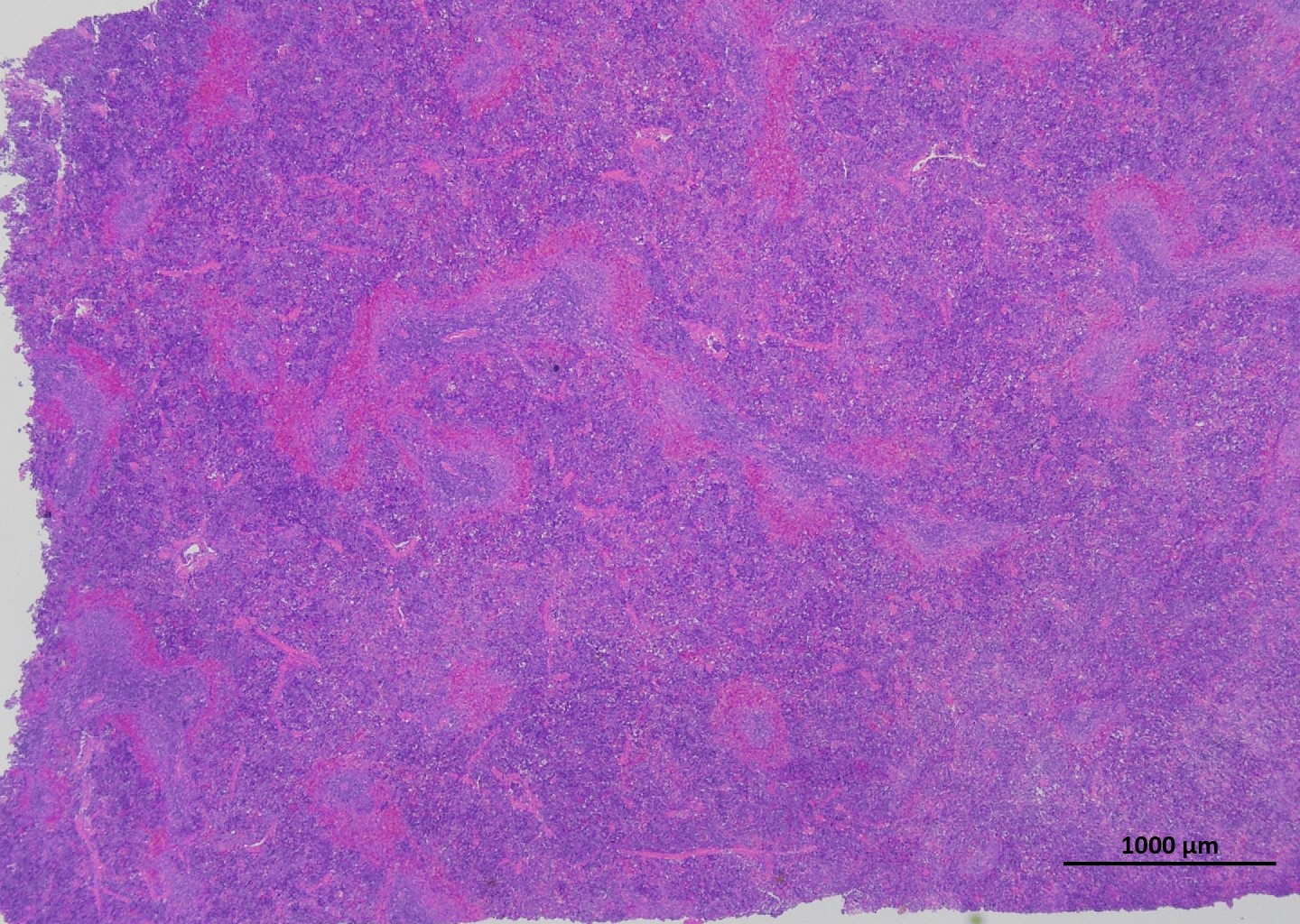

Supplement: Supplementary file 1 [file DataSheet_1.zip › HE/CA-M-20.jpg]

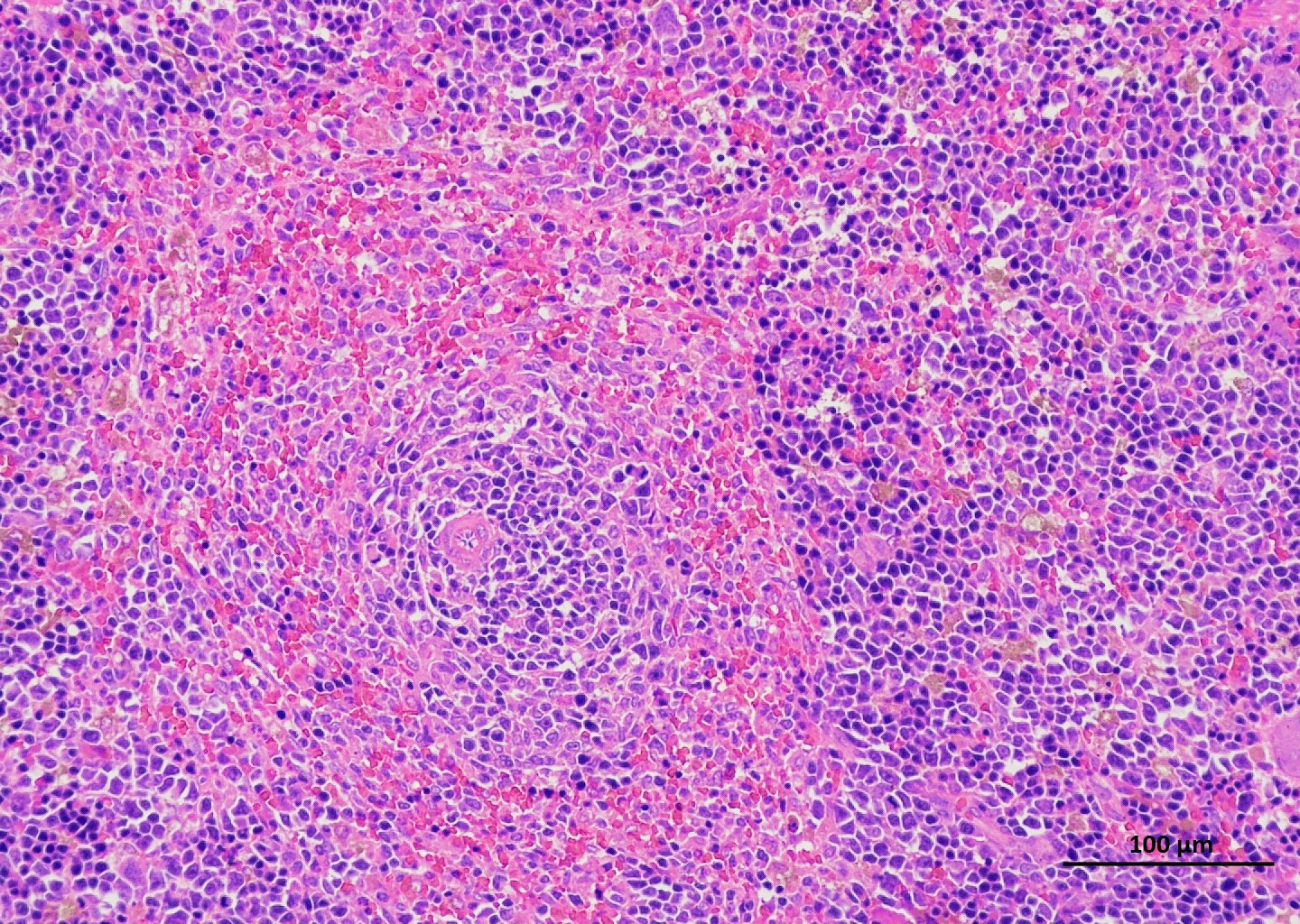

Supplement: Supplementary file 1 [file DataSheet_1.zip › HE/CA-M-200.jpg]

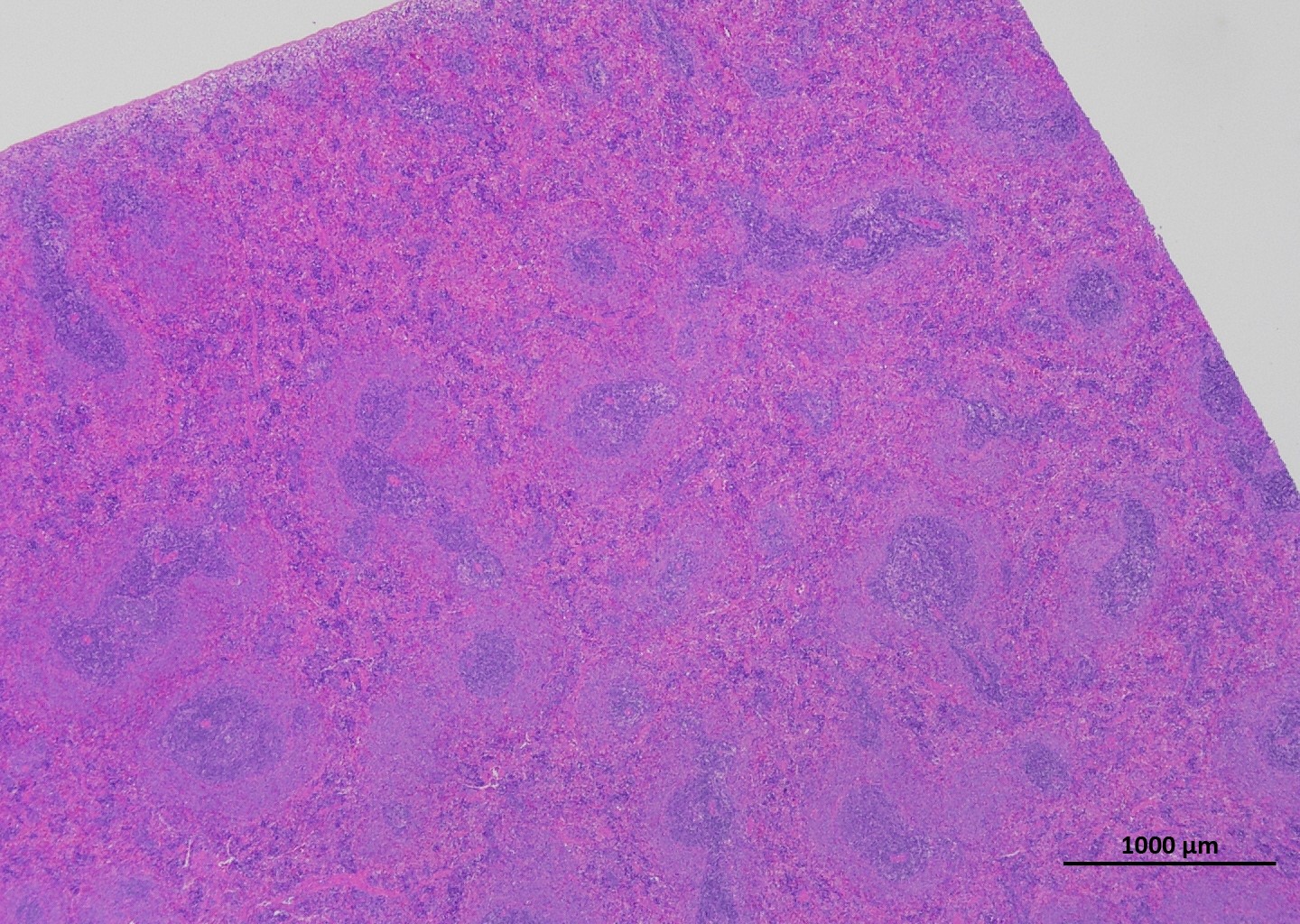

Supplement: Supplementary file 1 [file DataSheet_1.zip › HE/Control-20.jpg]

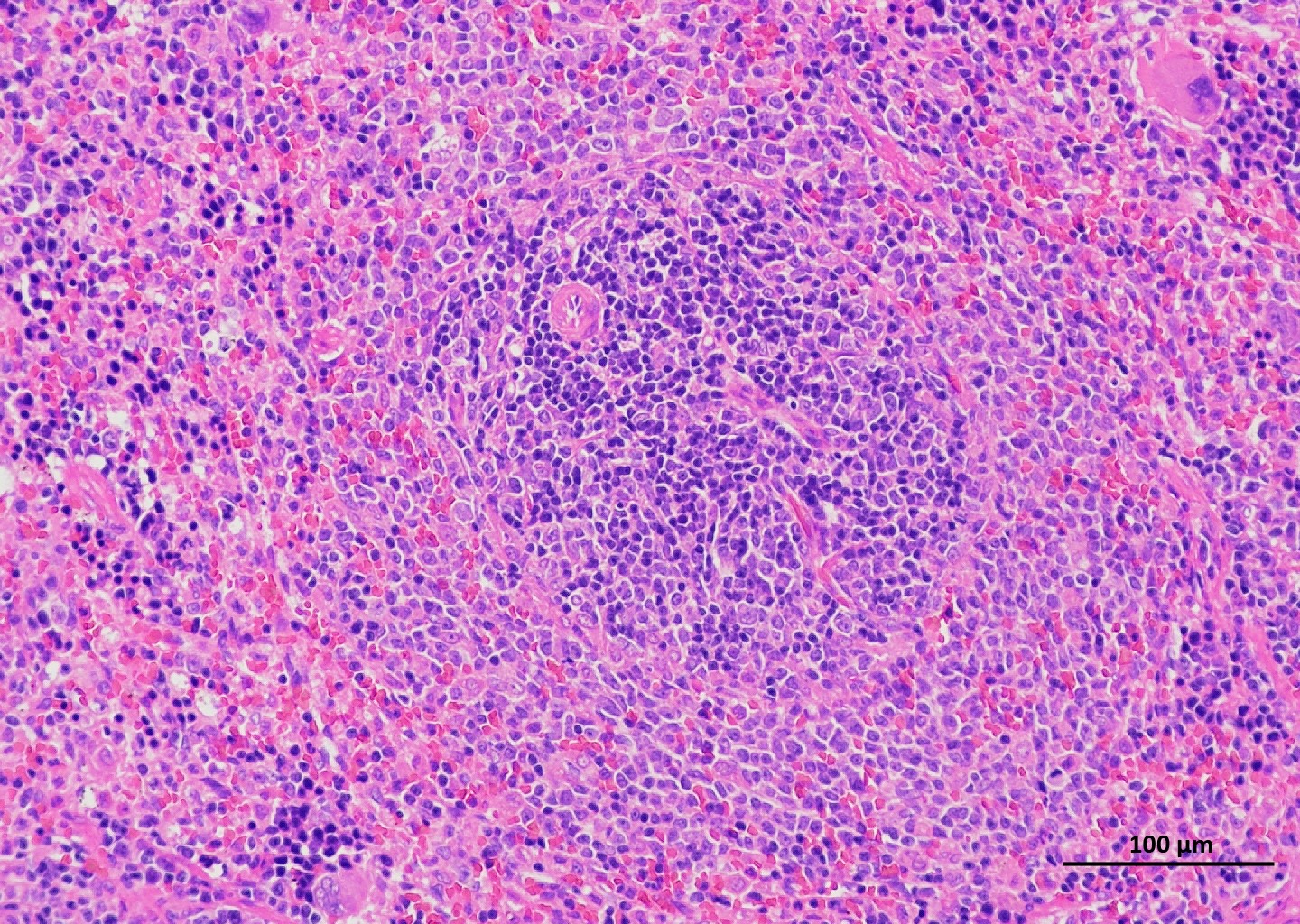

Supplement: Supplementary file 1 [file DataSheet_1.zip › HE/Control-200.jpg]

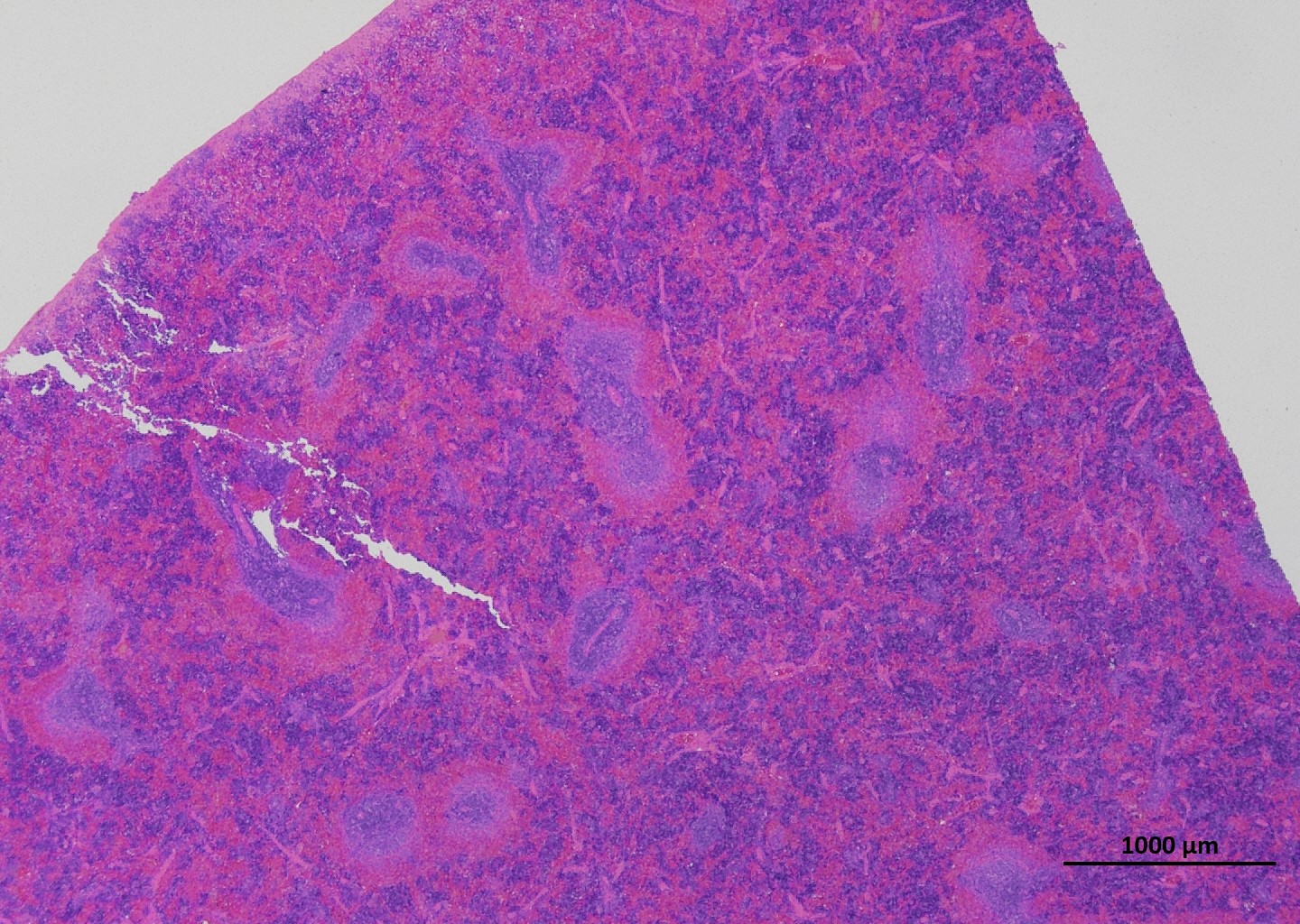

Supplement: Supplementary file 1 [file DataSheet_1.zip › HE/Model-20.jpg]

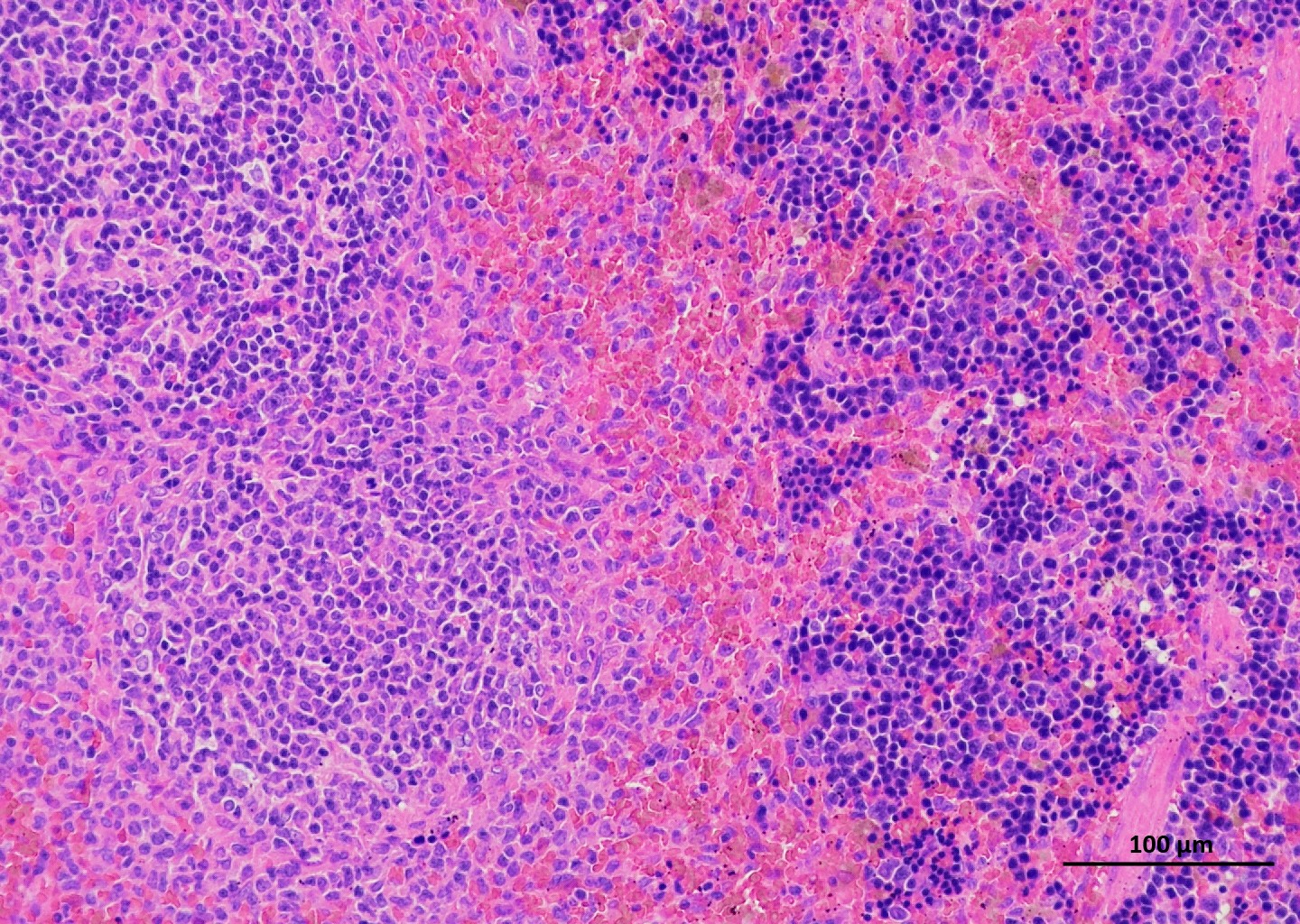

Supplement: Supplementary file 1 [file DataSheet_1.zip › HE/Model-200.jpg]
